# Time- and dose dependent actions of cardiotonic steroids on transcriptome and intracellular content of Na^+^ and K^+^: a comparative analysis

**DOI:** 10.1038/srep45403

**Published:** 2017-03-27

**Authors:** Elizaveta A. Klimanova, Artem M. Tverskoi, Svetlana V. Koltsova, Svetlana V. Sidorenko, Olga D. Lopina, Johanne Tremblay, Pavel Hamet, Leonid V. Kapilevich, Sergei N. Orlov

**Affiliations:** 1 Faculty of Biology, M. V. Lomonosov Moscow State University, Moscow, 119234, Russia; 2 Research Centre, University of Montreal Hospital (CRCHUM), Montreal, H2X 0A9, Canada; 3 National Research Tomsk State University, Tomsk, 634050, Russia

## Abstract

Recent studies demonstrated that in addition to Na^+^,K^+^-ATPase inhibition cardiotonic steroids (CTSs) affect diverse intracellular signaling pathways. This study examines the relative impact of [Na^+^]_i_/[K^+^]_i_-mediated and -independent signaling in transcriptomic changes triggered by the endogenous CTSs ouabain and marinobufagenin (MBG) in human umbilical vein endothelial cells (HUVEC). We noted that prolongation of incubation increased the apparent affinity for ouabain estimated by the loss of [K^+^]_i_ and gain of [Na^+^]_i_. Six hour exposure of HUVEC to 100 and 3,000 nM ouabain resulted in elevation of the [Na^+^]_i_/[K^+^]_i_ ratio by ~15 and 80-fold and differential expression of 258 and 2185 transcripts, respectively. Neither [Na^+^]_i_/[K^+^]_i_ ratio nor transcriptome were affected by 6-h incubation with 30 nM ouabain. The 96-h incubation with 3 nM ouabain or 30 nM MBG elevated the [Na^+^]_i_/[K^+^]_i_ ratio by ~14 and 3-fold and led to differential expression of 880 and 484 transcripts, respectively. These parameters were not changed after 96-h incubation with 1 nM ouabain or 10 nM MBG. Thus, our results demonstrate that elevation of the [Na^+^]_i_/[K^+^]_i_ ratio is an obligatory step for transcriptomic changes evoked by CTS in HUVEC. The molecular origin of upstream [Na^+^]_i_/[K^+^]_i_ sensors involved in transcription regulation should be identified in forthcoming studies.

Na^+^,K^+^-ATPase, a heterodimer consisting of catalytic α- and regulatory β-subunits, plays a key role in the maintenance of electrochemical gradients of monovalent cations across the plasma membrane (high intracellular potassium, [K^+^]_i_ versus low intracellular sodium, [Na^+^]_i_) in all types of nucleated animal cells studied so far. Since the seminal report of Jens Skou^1^, it has been demonstrated that cardenolides and other cardiotonic steroids (CTSs) inhibit Na^+^,K^+^-ATPase via their interaction with ubiquitous α1- and tissue-specific α2-α4 subunits that, in turn, leads to dissipation of Na^+^ and K^+^ transmembrane gradients and modulation of diverse [Na^+^]_i_,[K^+^]_i_-dependent cell functions, such as electrical membrane potential, cell volume, transepithelial movement of salt and osmotically-obliged water, and Na^+^ symport with glucose, amino acids, nucleotides, etc.^2^,^3^,^4^,^5^. Besides plant-derived cardenolides, other members of the CTS superfamily, bufadienolides, have been isolated from amphibians. Members of the cardenolide and bufadienolide families, ouabain and marinobufagenin (MBG), respectively, were identified in humans and are considered as endogenous CTSs involved in hypertension and others volume-expanded disorders (for comprehensive review, see refs 6 and 7).

During the last two decades, several research groups have presented data suggesting that along with canonical [Na^+^]_i_/[K^+^]_i_-mediated cellular processes, CTSs may affect cells independently of suppression of the Na^+^/K^+^ pump, i.e. might be considered as a novel class of steroid hormones. The evidence for [Na^+^]_i_/[K^+^]_i_-independent signals triggered by CTS are based on the observations listed below. *First*, in several types of cells, low doses of cardenolides and bufadienolides trigger membrane trafficking, proliferation, and [Ca^2+^]_i_ oscillation and affect signaling pathways terminated by activation of mitogen-activated protein kinases (MAPK) and protein kinase B or Akt (for reviews, see refs 6,8 and 9). *Second*, amino acid sequences that are crucial for interaction with Scr kinase, inositol 1,4,5-triphosphate receptor and several other proteins involved in signal transduction and biomembrane structure have been identified within Na^+^,K^+^-ATPase α-subunits^10^. *Third*, unlike ouabain, full-scale inhibition of the Na^+^,K^+^-ATPase in K^+^-free medium did not trigger the death of Madin-Darby canine kidney (MDCK) cells^11^,^12^ and endothelial cells from porcine aorta^13^. *Fourth*, in spite of the same inhibitory action on the Na^+^,K^+^-ATPase, distinct CTSs trigger different cellular responses^14^ and differentially affect the conformation of purified α1-Na^+^,K^+^-ATPase^15^.

More recently, we demonstrated that in several mammalian cell types including human umbilical vein endothelial cells (HUVEC), transcriptomic changes triggered by long-lasting application of ouabain were mimicked by inhibition of Na^+^,K^+^-ATPase in K^+^-free medium, thus suggesting a [Na^+^]_i_/[K^+^]_i_-mediated mechanism of excitation–transcription coupling^16^. We designed this study to examine the relative impact of [Na^+^]_i_/[K^+^]_i_-mediated and -independent signaling in transcriptomic changes evoked by endogenous CTS. To achieve this goal, we compared dose- and time-dependent actions of ouabain and MBG on Na^+^ and K^+^ content and transcriptomic changes in HUVEC. We also employed K^+^-free medium as an alternative approach for Na^+^,K^+^-ATPase inhibition and elevation of the [Na^+^]_i_/[K^+^]_i_ ratio.

## Results

### Viability of CTS-treated cells

Previously, it was shown that prolonged incubation with high doses of CTSs resulted in the death of human endothelial cells^17^. Considering this, we examined dose- and time-dependent actions of CTSs on the survival of HUVEC. After 24-, 48-, and 72-h incubations, the cytotoxic action of ouabain estimated by cell detachment was observed at concentration more than 100, 30, and 10 nM, respectively, whereas after 6-h incubation the cells survived even in the presence of 3,000 nM ouabain (see Supplementary Fig. S1). The dose- and time-dependent pattern of the cytotoxic action of ouabain was further confirmed by chromatin cleavage assay (see Supplementary Table S1) and the measurement of caspase-3 activity (see Supplementary Table S2).

In additional experiments, we compared cell survival after 96-h incubation with low doses of ouabain and MBG. To escape degradation of CTSs during long-term incubation, we changed the incubation medium every 24 h. In this study, elevation of ouabain and MBG concentration to 3 and 30 nM, respectively, did not affect cell attachment, chromatin cleavage, and caspase-3 activity in 96 hr incubation whereas incubation with 10 nM ouabain increased these parameters (see Supplementary Table S3) thus indicating an accumulation of dead cells. Based on these results, for the measurement of intracellular content of monovalent cations and transcriptomic changes we selected the doses of CTSs having no impact on cell survival.

### Intracellular Na^+^ and K^+^ content

**Figure 1:**
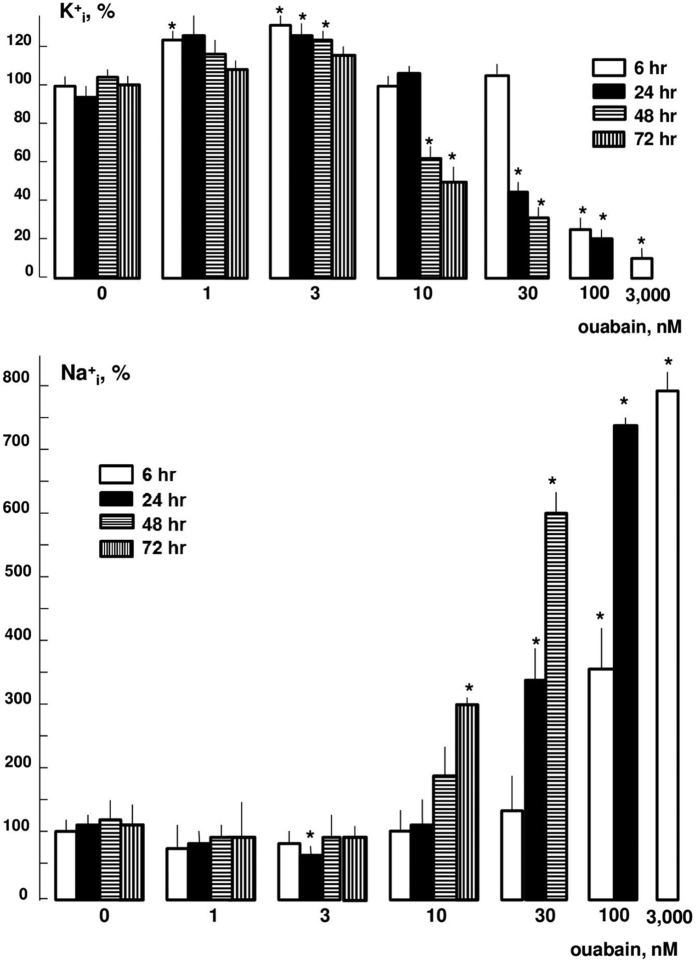
Dose-dependent actions of ouabain after 6-, 24-, 48-, and 72-h incubations on intracellular K^+^ and Na^+^ content in HUVEC. The K^+^_i_ and Na^+^_i_ content in cells for each time point in the absence of ouabain was taken as 100%. Means ± S.E. from three experiments performed in quadruplicates are shown. *p < 0.05 compared to ouabain-untreated cells at the selected incubation time.

Figure 1 shows that at concentrations of 1 and 3 nM ouabain increased K^+^_i_ and decreased Na^+^_i_ content by 20–30%. This observation is consistent with activation of Na^+^,K^+^-ATPase by low doses of CTSs detected by several research groups^18^,^19^. As predicted, at higher concentrations ouabain inhibited Na^+^,K^+^-ATPase and led to dissipation of the transmembrane gradient of monovalent cations. Importantly, the affinity for the inhibitory action of ouabain was increased with prolongation of incubation. Thus, after 6 h, the half-maximal elevation of [Na^+^]_i_ was detected at 100 nM ouabain, whereas after 24 and 72 h the same increment was detected at ouabain concentrations of 30 and 10 nM, respectively (Fig. 1). Then, we compared dose-dependent action of ouabain and MBG on intracellular Na^+^ and K^+^ content after 96-h incubation (see Supplementary Fig. S2). We found that at concentrations <1 nM, ouabain did not change these parameters, whereas at 3 nM it increased [Na^+^]_i_ and decreased [K^+^]_i_ by ~3 and 2-fold, respectively. In contrast to ouabain, MBG increased [Na^+^]_i_ and decreased [K^+^]_i_ at the concentration of 30 nM without any significant action on the [Na^+^]_i_/[K^+^]_i_ ratio at lower doses (see Supplementary Fig. S2). These results are consistent with data on attenuated sensitivity of the Na^+^,K^+^-pump to MBG compared to ouabain in neuronal cells^20^, purified human α1β1-, α2β1- and α3β1-Na^+^,K^+^-ATPase^21^, but contradicts to about the same efficacy of these CTSs obtained in rat mesenteric arteries^22^ and duck salt glands^15^.

### Transcriptomic changes

**Figure 2:**
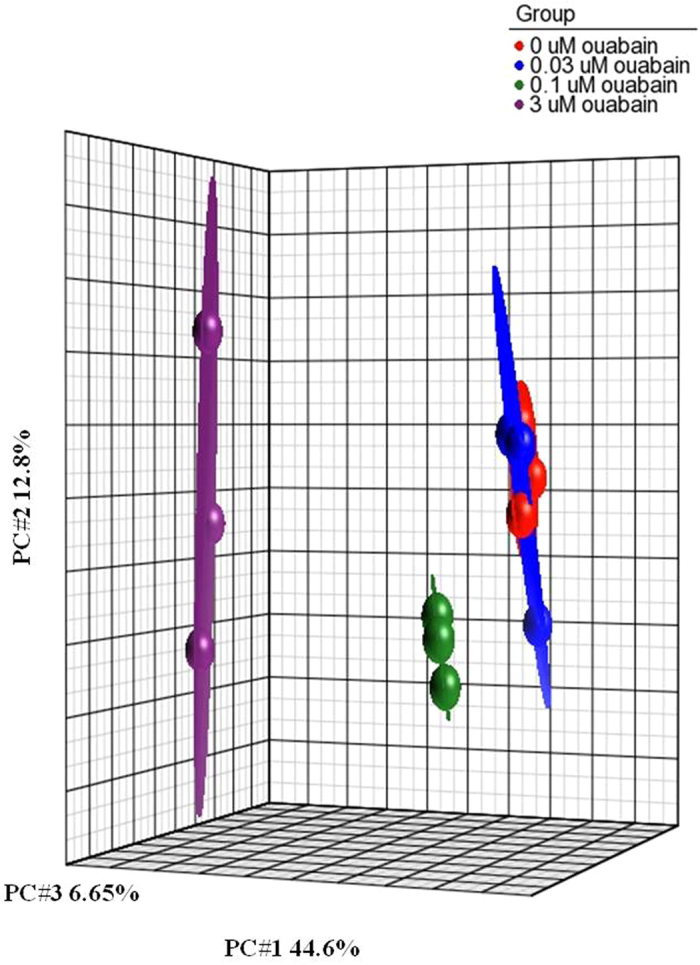
Principal component analysis (PCA) of the transcriptomes of HUVEC treated for 6 h in the absence or presence of 30, 100, or 3,000 nM ouabain. All experiments were repeated three times. Ellipsoids highlight portioning of samples based on the type of treatment. The principal components in three-dimensional graphs (PC#1, PC#2, and PC#3) represent the variability of gene expression level within the datasets. Each point on the PCA represents the gene expression profile of an individual sample. Samples that are near each other in the resulting three-dimensional plot have a similar transcriptome, while those that are further apart have dissimilar transcriptional profiles.

In the initial experiments, we examined the gene expression profile after 6-h exposure to 30, 100, and 3,000 nM ouabain. As noted above, at this time point 30 nM ouabain did not affect intracellular Na^+^ and K^+^ contents, whereas at concentrations of 100 and 3,000 nM it increased the [Na^+^]_i_/[K^+^]_i_ ratio by 16- and 86-fold, respectively (Fig. 1, Table 1). The transcriptomic data obtained in three independent experiments were normalized and then analyzed by principal component analysis (PCA) as described elsewhere^16^. This approach identified treatments with 100 and 3,000 nM ouabain but not with 30 nM ouabain as major sources of variability within datasets (Fig. 2).

Table 1 shows that in cells treated with 100 and 3,000 nM ouabain, i.e. at concentrations leading to elevation of the [Na^+^]_i_/[K^+^]_i_ ratio, the total numbers of transcripts whose expression was changed by more than 1.2-fold (p < 0.05) were 258 and 9277, respectively. These numbers roughly correspond to 1 and 30% of coding transcripts identified in the human genome. We did not observed any differentially expressed transcripts in cells subjected to 6-h incubation with 30 nM ouabain, i.e. at a concentration having no effect on intracellular Na^+^ and K^+^ contents. Importantly, among 258 differentially expressed transcripts seen after 6-h incubation with 100 nM ouabain, 197 transcripts were also subjected to altered expression in the presence of 3,000 nM ouabain. At least two hypotheses can be proposed to explain more pronounced transcriptomic changes detected with higher doses of ouabain. *First*, 3,000 nM ouabain leads to 5-fold higher increment of the [Na^+^]_i_/[K^+^]_i_ ratio compared to 100 nM ouabain (Table 1). *Second*, the rate of elevation of the [Na^+^]_i_/[K^+^]_i_ ratio is higher in cells treated with 3,000 nM ouabain compared to 100 nM ouabain.

Since CTSs have steroid structures, one might hypothesize that during long-term exposure they penetrate across the plasma membrane and affect gene transcription via a [Na^+^]_i_/[K^+^]_i_-independent interaction with nuclear mineralocorticoid receptors^5^,^23^. Considering this, we incubated cells for 96 h with 1 nM ouabain and 10 nM MBG lacking any effect on intracellular Na^+^ and K^+^ content, and with 3 nM ouabain and 30 nM MBG leading to elevation of the [Na^+^]_i_/[K^+^]_i_ ratio by ~7- and 3-fold, respectively (Table 2). Low doses of both CTSs had no impact on gene transcription, whereas elevation of the [Na^+^]_i_/[K^+^]_i_ ratio in the presence of 3 nM ouabain and 30 nM MBG resulted in appearance of 2185 and 1121 differentially expressed transcripts, respectively (Table 2).

**Figure 3:**
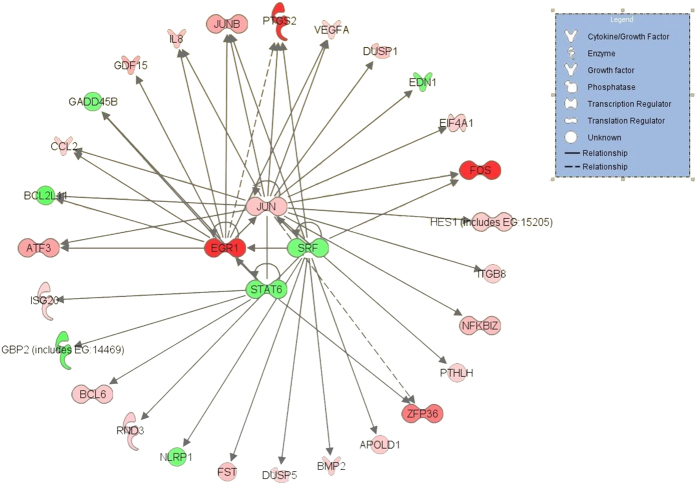
The signaling network possessing the highest score for association with differential expression of genes detected after 6-h incubation of HUVEC with 100 nM ouabain. The *p*-value and geometric fold change for each differentially expressed gene listed were overlaid onto a global molecular network developed from information within the IPA Knowledge Base. Genes are represented as nodes of various shapes to represent the functional category of the gene product as shown in the top corner, and the biological relationship between two nodes is represented as a line. Upregulated and downregulated genes are shown in pink and green, respectively.

Because genes are usually multifunctional, we limited their characterization to a few functional categories shown in Supplementary Tables S4–S7. Thus, the list of transcripts whose expression was changed by more than 2-fold under modest elevation of the [Na^+^]_i_/[K^+^]_i_ ratio occurring after 6-h exposure of HUVEC to 100 nM ouabain (Table 3) is abundant with genes involved in regulation of transcription and translation, including augmented expression of transcription factors FOS, EGR1, ZFP36, ATF3, and JUNB (see Supplementary Table S4). These genes probably play a key role in overall transcriptomic changes via upstream sensing of increased [Na^+^]_i_ and/or decreased [K^+^]_i_. Indeed, the Ingenuity database analysis (Fig. 3) shows that augmented expression of immediate response gene EGR1 may lead to altered expression of several other genes detected in this experiment, after 6 h of full-scale inhibition of the Na^+^,K^+^-ATPase by 3,000 nM ouabain (see Supplementary Tables S5 and S6), as well as after 96-h exposure to 3 nM ouabain and 30 nM MBG (Table 4 and Supplementary Tables S7) The transcriptomic changes presented in these tables include augmented expression of cyclooxygenase COX-2 (PTGS2), metallopeptidases (ADAMTS1, ADAMTS5, ADAMTS4, ADAMTS9), nuclear receptor subfamily 4 (NR4A1-NR4A3), and genes involved in regulation of cell growth, differentiation, and death (KIT ligand KITLG, Kruppel-like factor KLF4, growth differential factor GDF15, tumor necrosis factor ligand TNFSF9, thioredoxin interacting protein TXNIP, G_o_-S_2_ cell cycle transition switch controlling gene G0S2, cyclin-dependent kinase WEE1) (see Supplementary Tables S5–S7). The gene ontology analysis of the transcriptomic data is given in the Supplementary Tables S8–S11.

It is well-documented that protein synthesis is sharply inhibited in the absence of K^+^ via suppression of the translation elongation step, without any impact on ribosome subunit assembly (for review, see ref. 24). The molecular mechanisms of this phenomenon remains poorly understood. We observed ~3-fold attenuation of mRNA encoding eukaryotic translation initiation factor 5 (eIF5) (Table 3, see Supplementary Table S4) playing an ubiquitous role in protein synthesis by triggering GTP hydrolysis and mRNA translation^25^,^26^.

Supplementary Table S6 shows that 6-h elevation of the [Na^+^]_i_/[K^+^]_i_ ratio triggered by 3,000 nM ouabain causes ~7-fold attenuation of expression of EDN1, i.e. the gene encoding preproendothelin-1 that is proteolytically processed to the most powerful endothelium-derived vasoconstrictor endothelin-1 (ET-1), and ~10-fold elevation of the content of mRNA encoding ubiquitously derived vasodilator adrenomedulin (ADM) (see Supplementary Table S5). These results suggest that stimuli leading to dissipation of transmembrane gradients of monovalent cations in endothelial cells can lead to vasodilatation via disbalance in the secretion of these regulators of smooth muscle contraction. Additional experiments using advanced molecular biological and pharmacological approaches should be performed to dissect the relative impact of immediate response genes listed above in overall transcriptomic changes detected CTS-treated HUVEC.

### K^+^-free medium mimics the action of ouabain

To further explore the role of the [Na^+^]_i_/[K^+^]_i_ ratio in transcriptomic changes triggered by CTS, we employed qRT-PCR and compared the action of ouabain and K^+^-free medium on expression of three selected genes (*EGR1, PTGS2*, and *ATF3*). As predicted, Na^+^,K^+^-ATPase inhibition in K^+^-free medium led to drastic elevation of the intracellular Na^+^ content (Table 5, see Supplementary Fig. S3). Consistent with the data obtained by total genome scan (Table 3), the content of *EGR1, PTGS2*, and *ATF3* was increased after 6-h exposure to 100 nM ouabain by ~6, 6, and 2-fold, respectively. We observed a highly significant positive correlation between the increment of expression of these genes in the presence of ouabain and in K^+^-free medium (see Supplementary Fig. 3S).

## Discussion

Our results show that transcriptomic changes in endothelial cells treated with ouabain and marinobufagenin, i.e. two CTSs detected in humans and other mammalian species, are accompanied by the gain of Na^+^_i_ and loss of K^+^_i_ thus suggesting a key role of [Na^+^]_i_/[K^+^]_i_-mediated rather than [Na^+^]_i_/[K^+^]_i_-independent signaling. This hypothesis i**s** based on three major observations. *First*, neither the transcriptome nor the [Na^+^]_i_/[K^+^]_i_ ratio were affected by 6-h exposure of HUVEC to 30 nM ouabain having no effect on intracellular Na^+^ and K^+^ content, whereas elevation of ouabain concentration to 100 nM resulted in elevation of the [Na^+^]_i_/[K^+^]_i_ ratio by ~15-fold and appearance of 258 differentially expressed transcripts (Table 1). *Second*, The 96-h incubation with 3 nM ouabain or 30 nM MBG elevated the [Na^+^]_i_/[K^+^]_i_ ratio by ~14 and 3-fold and led to differential expression of 880 and 484 transcripts, respectively. These parameters were not changed after 96-h incubation with 1 nM ouabain or 10 nM MBG (Table 2). *Third*, we observed a positive correlation between the increment of gene expression in the presence of ouabain and in K^+^-free medium (Table 5 and Supplementary Fig. 3S).

It has been proposed that a signaling cascade triggered by the interaction of the Na^+^,K^+^-ATPase with membrane-associated nonreceptor tyrosine kinase Src is independent of any changes in intracellular Na^+^ and K^+^ concentrations and leads to activation of a diverse signaling cascade including phosphorylation of MAPK. Indeed, initial publications reported augmented tyrosine phosphorylation at ouabain concentrations having no significant action on ^86^Rb^+^ influx and intracellular Na^+^ content^27^,^28^,^29^. Later on, using human umbilical artery endothelial cells (HUAEC), Saunders and Scheiner-Bobis demonstrated that ouabain concentrations below 10 nM stimulated ^86^Rb^+^ uptake by 15–20%, increased cell growth by ~50%, phosphorylated EEK1/2 MAPK, and augmented the content of endothelin 1 mRNA (EDN1) by ~20% 30. Recently, we confirmed the proliferative action of low doses of ouabain using HUVEC; we also demonstrated that in these cells the proliferative action of ouabain is mediated by activation of the Na^+^,K^+^-ATPase and attenuation of the [Na^+^]_i_/[K^+^]_i_ ratio^31^. However, we noted that 6-h application of 3,000 nM ouabain causes ~7-fold attenuation rather than elevation of EDN1 expression (see Supplementary Table S6). Additional experiments should be performed to clarify whether or not these differences contribute to the vascular bed-specific properties of endothelial cells^32^ and to identify the role MAPK-mediated signaling in transcriptomic changes triggered by CTS.

The structural similarity of CTSs and steroid hormones suggests that along with Na^+^,K^+^-ATPase α-subunit, long-term exposure to low doses of CTSs can affect gene expression via their interaction with canonical transcription regulators such as xenobiotic-sensing nuclear receptor and receptors of mineralocorticosteroids^5^,^33^. Considering this, we increased the incubation time with ouabain to 96 h. We found that 96-h exposure to 3 nM ouabain or 30 nM MBG resulted in ~6-fold elevation of the [Na^+^]_i_/[K^+^]_i_ ratio by 6 and 3-fold (see Supplementary Fig. S2) and appearance of ~2,000 and 1,000 differentially expressed transcripts, respectively (Table 2). Importantly, among 79 genes whose expression was changed after 96-h incubation with 3 nM ouabain, 50 genes were also subjected to up- or downregulation by 30 nM MBG. Neither intracellular content of monovalent cations nor gene expression profile was affected by 96-h incubation of HUVEC with 1 nM ouabain or 10 nM MBG (see Table 2 and Supplementary Fig. S2).

Several research teams have reported altered expression of genes in cells treated by low doses of CTSs. For example, Li and coworkers found that 12-h incubation of bovine vascular smooth muscle cells with 10^−12^ M ouabain augmented c-myc mRNA content by 25–30% 34. Ren and coworkers observed that 2-h exposure of HUVEC to 0.3 but not to 1.8 nM ouabain decreased the content of IkB by 4-fold^35^. Using 2D-electrophoresis, Qiu *et al*. identified five proteins whose expression was changed after 24-h incubation of HUVEC with 10 nM ouabain by 2–3-fold^36^. It should be emphasized, however, that intracellular Na^+^ and K^+^ content was not measured in the above-cited studies at the time points used for the analysis of gene expression. This comment becomes important because cell threshold for elevation of the [Na^+^]_i_/[K^+^]_i_ ratio by ouabain was decreased with prolongation of incubation time from 6 to 72 h by ~10-fold (Fig. 1). This phenomenon can be explained by the slow kinetics of ouabain binding with Na^+^,K^+^-ATPase at low concentrations documented in previous investigation^37^ as well as by time-dependent elevation of [Na^+^]_i_ and accumulation of Na^+^,K^+^-ATPase in CTS-sensitive E2 conformation possessing augmented affinity for CTSs.

The molecular origin of [Na^+^]_i_ /[K^+^]_i_ sensors involved in transcription regulation is still a mystery. *A priori*, elevation of the [Na^+^]_i_/[K^+^]_i_ ratio may effect transcription via increment in [Ca^2+^]_i_ caused by activation of the Na^+^_i_/Ca^2+^_o_ exchanger and/or Ca^2+^ influx via voltage-gated channels – a phenomenon termed excitation–transcription coupling^38^,^39^,^40^. To further examine the role of Ca^2+^_i_-mediated signaling, we employed Ca^2+^ chelators. Surprisingly, these compounds increased rather than decreased the number of differentially expressed transcripts in HUVEC exposed to 3,000 nM ouabain^16^. Investigating this intriguing phenomenon, we found that along with the canonical mechanism, extracellular Ca^2+^ chelators affect gene expression via elevation of plasma membrane permeability for Na^+^ and K^+^ 41. Thus, novel approaches should be developed for the estimation of the relative impact of [Ca^2+^]_i_-mediated signals and recently discovered Ca^2+^_i_-independent signaling pathways^42^,^43^ in transcription regulation by CTS.

## In conclusion

our data show that elevation of the [Na^+^]_i_/[K^+^]_i_ ratio is an obligatory step of transcriptomic changes in CTS-treated cells. Additional experiments should be performed to identify the molecular origin of upstream [Na^+^]_i_/[K^+^]_i_ sensors involved in [Na^+^]_i_/[K^+^]_i_-mediated excitation-transcription coupling as well as the relative impact of [Na^+^]_i_/[K^+^]_i_-independent signaling.

## Methods

### Cell culturing

Human umbilical vein endothelial cells (HUVEC) were purchased from Lonza (Walkersville, MD, USA) and passaged 4–8 times. The cells were cultured in complete endothelial cell growth medium-2 (EGM-2 BulletKit, CC3162, Lonza) containing 10% fetal bovine serum (FBS) and maintained in a humidified atmosphere with 5% CO_2_/balance air at 37 °C. To establish quiescence, the cells were incubated for 24 h in medium in which the concentration of FBS was reduced to 0.2%. Then, quiescent cells were washed with phosphate-buffered saline (PBS) and incubated for 96 h in EGM-2 containing 0.2% FBS and ouabain or MBG in the range from 1 to 3,000 nM. In some experiments, cells were incubated in Dulbecco’s Modified Eagle Medium (DMEM) or in K^+^-free medium DMEM (Invitrogen, Carlsbad, CA)

### Cell viability

The impact of CTSs on cell viability was studied by cell attachment assay as described in detail elsewhere^44^. Briefly, the HUVEC were seeded into 6-well plates and treated as indicated above. Then, the incubation medium was transferred into centrifugation tubes and combined with medium obtained after three washes in 2 ml of PBS. The detached cells were sedimented (1,000 g, 5 min) and washed once with 5 ml of PBS. The protein content of detached cells (*PR*_*de*t_) and cells attached to the plastic supports after three washes with 2-ml aliquots of medium W (*PR*_*att*_) was measured by a modified Lowry method. Total protein content (*PR*_*att*_ + *PR*_*det*_) was taken as 100%. In additional experiments, cell viability was assessed by measuring caspase-3 activity and chromatin cleavage. Caspase-3 activity in cells growing in 6-well plates was measured as the rate of the caspase-3 inhibitor (Ac-DEVD-CHO)-sensitive component of caspase-3 fluorescent substrate (DEVD-AMC, N-acetyl-Asp-Glu-Val-Asp-AMC) hydrolysis according to a previously described protocol^45^. To estimate chromatin fragmentation, HUVEC in 24-well dishes were supplied with DMEM containing serum and 0.1 μCi/ml [^3^H]-thymidine. After 24 h, they were washed twice with 2 ml of DMEM and incubated for 48 h in DMEM with serum and compounds as indicated in the figure and table legends. Then the medium was collected and centrifuged at 900 g for 10 min. Next, the supernatant was transferred for the measurement of radioactivity in a liquid scintillation spectrometer (fraction *F*_*1*_), and the cell pellet and cells remaining in the plates were treated for 15 min with ice-cold lysing buffer (10 mM EDTA, 10 mM Tris-HCl, 0.5% Triton X-100, pH 8.0). Then the cell lysates were combined, sedimented (12,000 rpm, 10 min), and the supernatant was transferred for radioactivity measurement (fraction *F*_*2*_). The remaining radioactivity from the pellets and wells was extracted with a 1% SDS/4 mM EDTA mixture (fraction *F*_*3*_). The relative content of intracellular chromatin fragments was determined as a percentage of total [^3^H]-labeled DNA: *F*_*2*_*/(F*_*1*_ + *F*_*2*_ + *F*_*3*_)^*–1*^ × *100%*. For more details, see ref. 45.

### Intracellular content of exchangeable K^+^ and Na^+^

was measured as the steady-state distribution of extra- and intracellular ^86^Rb and ^22^Na, respectively. Briefly, the cells were seeded in 24- or 12-well plates (^86^Rb and ^22^Na, respectively), treated up to 72 h in the presence or absence of CTS, transferred onto ice, and washed four times with 2 ml of ice-cold medium W containing 100 mM MgCl_2_ and 10 mM HEPES-Tris buffer (pH 7.4). The washing medium was aspirated, and the cells were lysed with 1% SDS and 4 mM EDTA solution. Radioactivity of the incubation media and cell lysates was quantified, and intracellular cation content was calculated as *A/am*, where *A* was the radioactivity of the samples (cpm), *a* was the specific radioactivity of ^86^Rb (K^+^) and ^22^Na in the medium (cpm/nmol), and *m* was the protein content. For more details, see ref. 16 and 45.

### RNA isolation

Total RNA was extracted from cells grown in 6-well plates using TRIzol^®^ reagent (Invitrogen, Carlsbad, CA) and purified with an RNeasy^®^ MinElute cleanup kit (Qiagen, Valencia, CA) following the manufacturers’ protocols. Only the RNA samples that had more than 7.0 RNA integrity number (RIN) and no detectable genomic DNA contamination were used for the subsequent gene array analyses. RNA quality was assessed using a 2100 Bioanalyzer (Agilent Technologies, Palo Alto, CA). Microarray experiments were performed with a GeneChip^®^ Human Gene 1.0 ST array detecting 28,869 gene products. Each gene was represented by approximately 26 probes along the entire length of the transcript (Affymetrix, Santa Clara, CA). Total RNA (100 ng for each sample) was processed with an Ambion^®^ WT Expression Kit (Invitrogen). This kit uses a reverse transcription priming method that specifically primes nonribosomal RNA, including both poly(A) and non-poly(A) mRNA, and generates sense-strand cDNA as the final product. The single-stranded cDNA (5.5 μg) was fragmented and labeled using the Affymetrix GeneChip^®^ WT Terminal Labeling Kit, and 2.0 μg of the resulting cDNA was hybridized on the chip.

### GeneChip expression analysis

The whole hybridization procedure was conducted with the Affymetrix GeneChip^®^ system according to the protocol recommended by the manufacturer. The hybridization results were evaluated with Affymetrix GeneChip^®^ Command Console Software (AGCC). The quality of the chips was determined using the Affymetrix Expression Console. Data analysis was performed with the Partek Genomics Suite (Partek, St. Louis, Missouri). The data were initially normalized by the Robust Multichip Average (RMA) algorithm, which uses background adjustment, quantile normalization, and summarization. Then normalized data were analyzed by principal component analysis (PCA)^46^ to identify patterns in the dataset and highlight similarities and differences among the samples. Major sources of variability identified within the dataset by PCA were used as grouping variabilities for analysis of variance (ANOVA) with n = 4 for each group of samples. The ensuing data were filtered to identify transcripts with statistically significant variation of expression among the groups that are modulated by at least 20%, with multiple testing correction by the false discovery rate (FDR). The calculated *p*-value and geometric fold change for each probe set identifier were imported into Ingenuity Pathway Analysis (IPA, Ingenuity Systems, http://www.ingenuity.com) to ascertain networks, biological functions, and their pathophysiological implications. Functional information on regulated genes was also obtained using PubMed and the cited publications.

### Real-time quantitative RT-PCR

We used qRT-PCR to measure the content of selected transcripts whose expression was increased after 6-h incubation with 100 nM ouabain by more than 2-fold. In these experiments, we employed Express SYBR GreenER qPCR Supermix kit (Invitrogen, Carlsbad, CA, USA) according to the manufacturer’s instructions. The reaction was carried out with a 7900HT Fast RT PCR system (Applied Biosystems, Foster City, CA). Primers were designed using Primer3Plus online software from consensus sequences provided by Affymetrix for each gene of interest. The relevant primer sequences were: *EGR1*-forward 5′CTTCAACCCTCAGGCGGACA3′; *EGR1*-reverse 5′GGAAAAGCGGCCAGTATAGGT3′; *PTGS2*-forward 5′CAGCCATACAGCAAATCCTTG3′; *PTGS2*-reverse 5′AATCCTGTCCGGGTACAATC3′; *ATF3*-forward 5′ATGATGCTTCAACACCCAGGC3′; *ATF3*-reverse 5′TTAGCTCTGCAATGTTCCTTC3′. β_2_ microglobulin mRNA expression was used to normalize and compare the expression values of genes of interest. The results were quantified by the ΔΔCt method with Excel Microsoft software.

### Statistical analysis

The comparison of data groups was performed by Student’s t-test and Pearson correlation coefficient. Transcriptomic data were analysed using WebGestalt^47^,^48^.

### Chemicals

Methyl-[^3^H]-thymidine was purchased from ICN Biomedicals, Inc. (Irvine, CA); ^22^NaCl and ^86^Rb were obtained from Perkin Elmer (Waltham, MA) and Isotope (Russia). Marinobufagenin was kindly provided by Dr. A. Y. Bagrov (NIH, Baltimore). The remaining chemicals were from Gibco BRL (Gaithersburg, MD), Calbiochem (La Jolla, CA), Sigma (St. Louis, MO), and Anachemia (Montreal, QC).

## Additional Information

**How to cite this article**: Klimanova, E. A. *et al*. Time- and dose dependent actions of cardiotonic steroids on transcriptome and intracellular content of Na^+^ and K^+^: a comparative analysis. *Sci. Rep.* **7**, 45403; doi: 10.1038/srep45403 (2017).

**Publisher's note:** Springer Nature remains neutral with regard to jurisdictional claims in published maps and institutional affiliations.

## Supplementary Material

Supplementary Information

## Figures and Tables

**Table 1:** Dose-dependent action of 6-h incubation with ouabain on the [Na^+^]_i_/[K^+^]_i_ ratio and transcriptome of HUVEC.

Parameter	Ouabain, nM		
	*30*	*100*	*3,000*
[Na^+^]_i_/[K^+^]_i_	1.19	14.97*	78.13*
Number of upregulated transcripts	ND	124	4117
Maximal fold of activation		8.71	197.05
Number of downregulated transcripts	ND	134	5160
Maximal fold of inhibition		2.85	15.75

Upregulated and downregulated transcripts were defined as transcripts whose expression was increased or decreased by more than 1.2-fold (p < 0.05). The [Na^+^]_i_/[K^+^]_i_ rat_i_o was calculated from data presented in Fig. 1 and taken in the absence of ouabain as 1.00. *p < 0.001 compared to the [Na^+^]_i_/[K^+^]_i_ ratio after 6-h incubation in the absence of ouabain. ND – differentially expressed transcripts were not detected.

**Table 2:** Dose-dependent action of 96-h incubation with ouabain and MBG on the ratio [Na^+^]_i_/[K^+^]_i_ and transcriptome of HUVEC.

Parameter	Ouabain, nM		MBG, nM	
	1	3	10	30
[Na^+^]_i_/[K^+^]_i_	1.01	13.79**	1.06	2.95*
Number of upregulated transcripts	ND	880	ND	484
Maximal fold of activation		33.28		21.43
Number of downregulated transcripts	ND	1305	ND	637
Maximal fold of inhibition		2.71		3.13

Upregulated and downregulated transcripts were defined as transcripts whose expression was increased or decreased by more than 1.2-fold (p < 0.05). The [Na^+^]_i_/[K^+^]_i_ rat_i_o was calculated from data presented in the Supplementary Fig. S2 and taken in the absence of ouabain as 1.00. *, **p < 0.01 and <0.001 compared to the [Na^+^]_i_/[K^+^]_i_ ratio after 96-h incubat_i_on in the absence of ouabain. ND – differentially expressed transcripts were not detected.

**Table 3:** Genes whose expression is modulated by 100 nM ouabain after 6-h incubation by more than 2-fold.

Gene symbol, title	Affymetrix ID	Fold of modulation/*p*-value
**FOS**//FBJ murine osteosarcoma viral oncogene homolog	7975779	6.07/1.39E-03
**EGR1**//early growth response 1	8108370	5.87/2.08E-04
**PTGS2**//prostaglandin-endoperoxide synthase 2	7922976	5.86/6.23E-05
**ADAMTS1**//ADAM metallopeptidase with thrombospondin type 1	8069676	4.16/6.23E-05
**ZFP36**//zinc finger protein 36, C3H type, homolog (mouse)	8028652	3.77/2.40E-03
**ATF3**//activating transcription factor 3	7909610	2.60/4.17E-03
**JUNB**//jun B protooncogene	8026047	2.49/1.82E-03
**MIR31**//microRNA 31	8160439	2.22/3.50E-03
**GDF15**//growth differentiation factor 15	8027002	2.22/1.52E-03
**C2orf61**//chromosome 2 open reading frame 61	8052004	2.20/2.26E-04
**KITLG**//KIT ligand	7965322	2.19/7.61E-04
**C7orf53**//chromosome 7 open reading frame 53	8135532	2.05/1.25E-03
**RPL27A**//ribosomal protein L27a	7938295	2.03/4.17E-03
**EIF5**//eukaryotic translation initiation factor 5	7977058	−2.84/4.53E-04
**MIR216A**//microRNA 216a	8052374	−2.08/8.99E-05
**MIR27B**//microRNA 27b	8156571	−2.02/3.50E-03

**Table 4:** Genes whose expression is modulated by 3 nM ouabain and 30 nM MBG after 96-h incubation.

Gene symbol	Affymetrix ID	*3 nM ouabain, fold of modulation/p-value*	*30 nM MBG, fold of modulation/p-value*
FOS	7975779	33.28/8.84E-07	13.29/4.84E-06
ZFP36	8028652	20.36/9.39E-07	6.09/7.00E-06
EGR1	8108370	15.33/3.86E-05	21.43/6.11E-08
ADAMTS1	8069676	13.04/8.84E-07	5.01/4.04E-04
PTGS2	7922976	11.36/1.38E-06	9.02/4.46E-06
EGR2	7933872	8.09/9.39E-07	10.19/6.06E-07
ATF3	7909610	7.39/6.40E-06	8.13/5.14E-06
ARRDC4	7986350	5.62/2.45E-06	NC
NR4A1	7955589	5.39/2.13E-05	7.29/3.30E-06
NR4A3	8156848	5.38/2.09E-05	6.00/5.25E-05
STC1	8149825	5.35/4.84E-04	NC
JUNB	8026047	5.06/3.43E-06	1.53/5.87E-03
KLF4	8163002	4.70/3.66E-05	3.03/1.21E-04
DUSP1	8115831	4.52/3.32E-06	6.09/3.33E-05
ADAMTS5	8069689	4.43/2.58E-05	2.16/2.01E-03
NR4A2	8055952	4.15/1.28E-04	2.99/4.56E-04
SNAI1	8063382	3.87/8.20E-06	NC
HEY1	8151457	3.75/1.21E-05	1.84/9.12E-03
SOCS3	8018864	3.75/7.12E-06	NC
VTRNA1–3	8108631	3.64/5.49E-05	2.76/3.12E-04
JUN	7916609	3.17/6.40E-06	4.72/3.60E-05
KITLG	7965322	3.05/3.99E-05	1.44/8.66E-03
C7orf53	8135532	3.00/6.76E-04	1.33/8.66E-03
EGR3	8149720	2.98/1.48E-04	3.07/3.09E-04
GDF15	8027002	2.98/9.39E-07	NC
IDI2	7931748	2.87/1.55E-03	NC
BAMBI	7926875	2.80/4.30E-04	1.29/1.68E-02
EFNB2	7972713	2.79/1.44E-05	1.52/6.33E-03
GEM	8151816	2.77/6.90E-04	2.71/7.67E-04
ABCG2	8101675	2.76/2.27E-06	NC
SNORD3A	8005547	2.72/2.03E-05	NC
FAM71A	7909624	2.66/1.36E-04	1.70/4.02E-04
IER2	8026163	2.65/8.00E-05	1.54/9.03E-03
HIST2H2BE	7919637	2.60/1.13E-04	1.67/7.66E-04
INSIG1	8137526	2.58/1.44E-05	1.25/9.33E-03
PROX1	7909681	2.52/2.45E-06	2.70/5.01E-04
HEY2	8121850	2.52/1.32E-04	2.07/9.81E-04
CA8	8150978	2.50/1.28E-03	NC
HIST1H1T	8124402	2.48/7.76E-03	1.32/8.08E-03
TNFSF9	8025053	2.47/1.45E-04	3.71/9.91E-05
DLL4	7982854	2.46/1.30E-05	NC
PTHLH	7962000	2.39/8.00E-05	NC
APOLD1	7954055	2.36/4.48E-04	NC
GUCY1B3	8097973	2.34/1.01E-05	NC
IGFBP5	8058857	2.28/8.16E-03	1.60/4.00E-03
SPRY4	8114797	2.28/2.56E-03	1.79/2.83E-03
BCL6	8092691	2.27/2.27E-06	NC
HIST2H4A	7905067	2.24/1.01E-04	1.88/4.31E-03
KCNJ2	8009502	2.24/8.00E-05	2.00/7.01E-04
TSC22D2	8083324	2.24/6.58E-05	NC
DDIT3	7964460	2.23/1.06E-03	2.78/3.33E-04
C2orf61	8052004	2.23/1.61E-03	NC
HIST1H2BG	8124423	2.19/7.34E-03	NC
HES1	8084880	2.19/2.45E-06	2.21/4.41E-04
ELMOD1	7943562	2.18/1.25E-04	NC
MAFB	8066266	2.16/9.95E-04	1.89/2.08E-03
HIST1H2BD	8117382	2.13/2.18E-03	NC
SNORD75	7922416	2.11/6.89E-04	−1.39/8.88E-03
ADAMTS9	8088560	2.10/1.30E-05	2.09/7.51E-04
ADAMTS4	7921821	2.09/2.67E-04	1.97/8.31E-04
KLF5	7969414	2.07/6.49E-04	1.62/1.51E-03
HIST2H2AA3	7905079	2.05/1.30E-04	NC
NEFM	8145361	2.04/6.82E-03	−1.25/6.77E-03
PCDH17	7969330	2.04/1.13E-04	NC
TXNIP	7904726	2.03/1.30E-05	2.22/6.89E-04
FJX1	7939365	2.03/2.41E-02	−1.33/9.38E-03
BHLHE40	8077441	2.03/1.80E-05	NC
ID2	8040103	2.02/2.60E-03	1.73/2.21E-05
G0S2	7909441	2.02/3.87E-04	2.06/7.66E-05
IDI1	7931754	2.01/1.39E-04	1.21/4.61E-03
MIRLET7C	8067944	−2,03/3.36E-03	NC
DTL	7909568	−2.05/8.00E-05	NC
KIAA1370	7988970	−2.07/4.94E-06	1.22/2.99E-03
FAM111B	7940147	−2.18/2.72E-03	−1.59/2.27E-03
PDK4	8141094	−2.32/4.18E-05	−1.48/8.72E-04
TRA2A	8138592	−2.40/3.39E-04	NC
EIF5	7977058	−2.52/6.54E-05	−2.59/4.72E-05
FBXO32	8152703	−2.71/1.95E-04	−1.73/3.70E-04

The table lists transcripts whose expression was changed in ouabain-treated cells by more than 2.0-fold.

**Table 5:** Effect of ouabain and K^+^-free medium on intracellular Na^+^ and the content of mRNA encoding EGR1, PTGS2, and ATF3.

Incubation medium	Na^+^_i_ content, nmol/mg protein	mRNA content, relative units		
		EGR1	PTGS2	ATF3
Control (DMEM)	84.0 ± 12.3	1.00	1.00	1.00
Ouabain, 100 nM	511 ± 52	7.34 ± 0.20	5.96 ± 0.09	2.14 ± 0.07
K^+^-free DMEM	843 ± 77	8.16 ± 0.17	6.51 ± 0.11	3.17 ± 0.16

Cells were incubated in control and K^+^-free medium as well as in the presence of 100 nM ouabain for 6 h. mRNA content was estimated by RT-qPCR and was taken in control medium as 1.00. Means ± S.E. from four experiments performed in triplicates (Na^+^ content) and duplicates (mRNA content) are shown.
