# Supplementary material for: Time- and dose dependent actions of cardiotonic steroids on transcriptome and intracellular content of Na+ and K+: a comparative analysis
Source: Sci Rep. 2017 Mar 27;7:45403. doi: 10.1038/srep45403 (PMC5366943; doi:10.1038/srep45403)
Supplement: Supplementary Information [file srep45403-s1.pdf]

Time- and dose dependent actions of cardiotonic steroids on transcriptome and intracellular content of Na^+ and K^+: a comparative analysis

Elizaveta A. Klimanova[1], Artem M. Tverskoi[1], Svetlana V. Koltsova[1], Svetlana V. Sidorenko[1], Olga D. Lopina[1], Johanne Tremblay[2], Pavel Hamet[2], Leonid V. Kapilevich[3] and Sergei N. Orlov[1,3]*

[1] Faculty of Biology, M. V. Lomonosov Moscow State University, Moscow, 119234, Russia
[2] Research Centre, University of Montreal Hospital (CRCHUM), Montreal, H2X 0A9, Canada
[3] National Research Tomsk State University, Tomsk, 634050, Russia
*sergeinorlov@yandex.ru

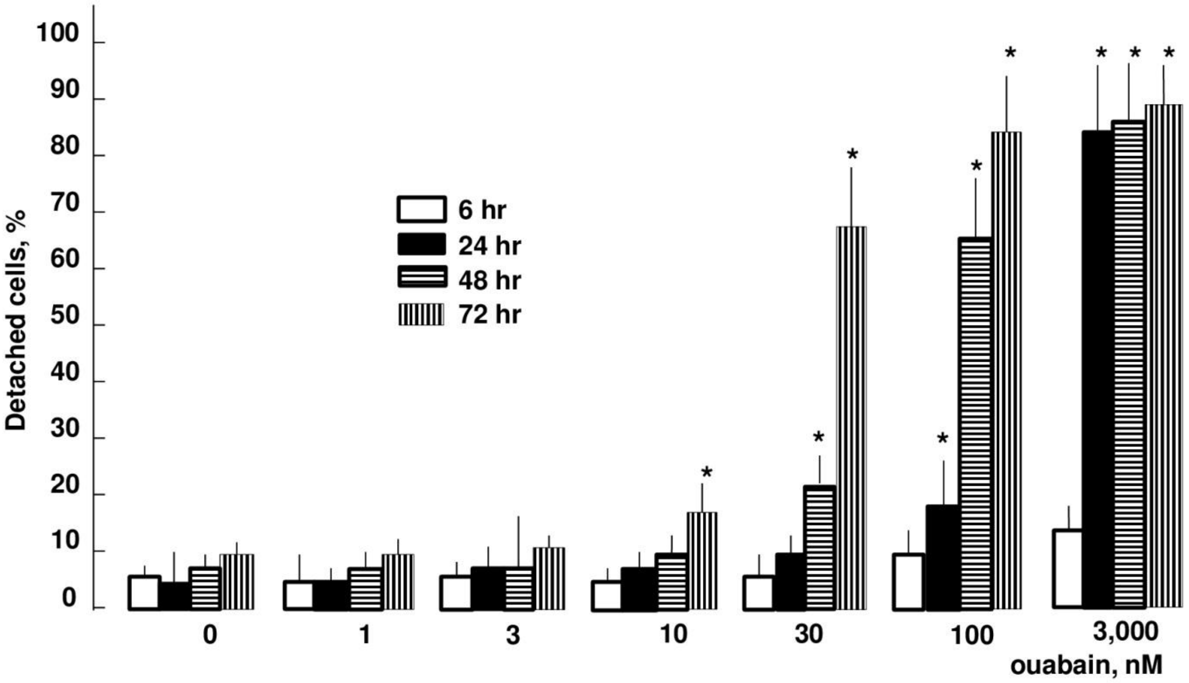

Figure S1. Dose-dependent actions of ouabain on viability of HUVEC after 6-, 24-, 48-, and 72-h incubations estimated by the cell-detachment assay. Total protein content in attached and detached cells was taken as 100%. Means ± S.E. from three experiments performed in quadruplicates are shown. * $p<0.05$ compared to ouabain-untreated cells at the selected incubation time.

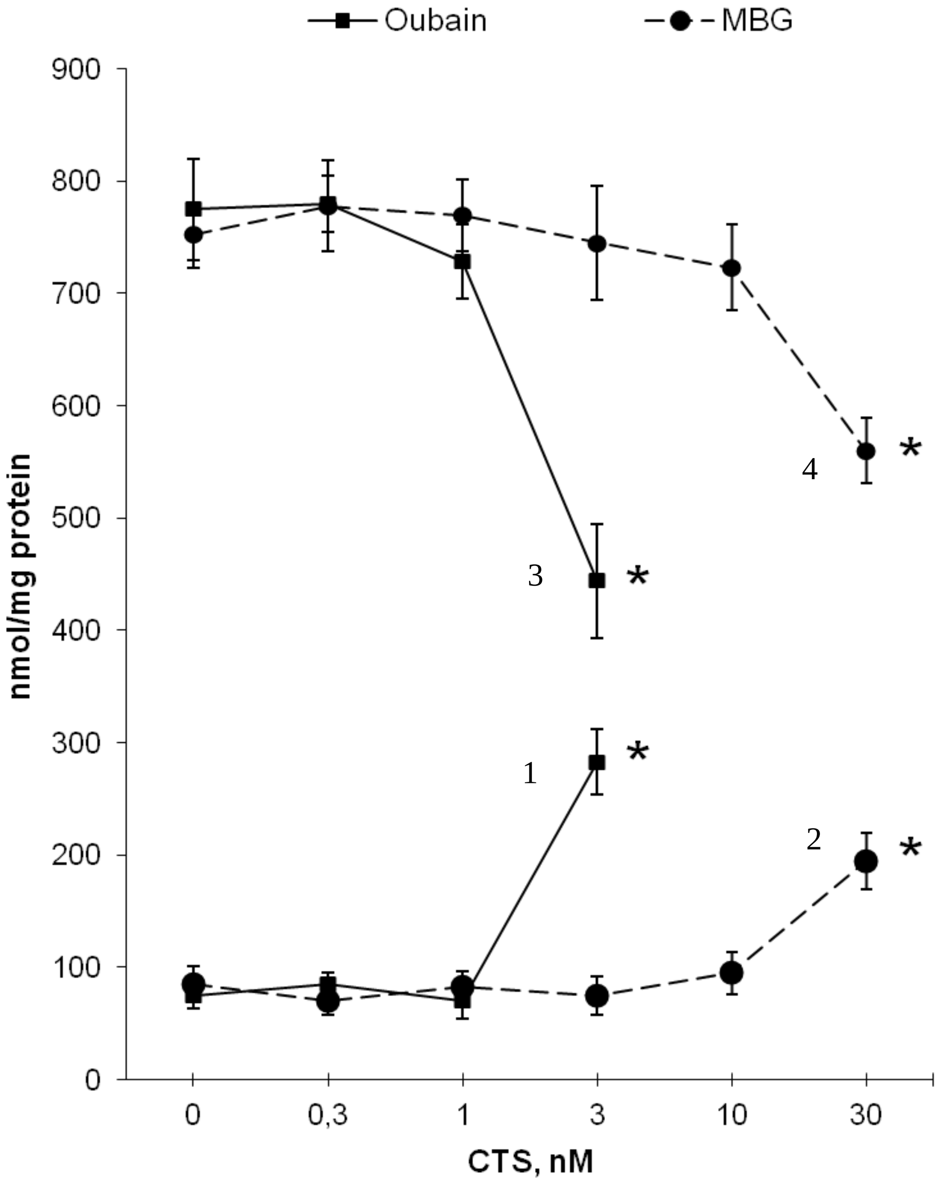

Figure S2. Dose-dependent actions of 96-h incubation with ouabain (curves 1 and 3) and MBG (curves 2 and 4) on intracellular Na^+ content (curves 1 and 2) and K^+ (curves 3 and 4) in HUVEC. The incubation medium was replaced every 24 h. Means ± S.E. from experiments performed in quadruplicates are shown. * $p<0.01$ compared control (CTS-free medium).

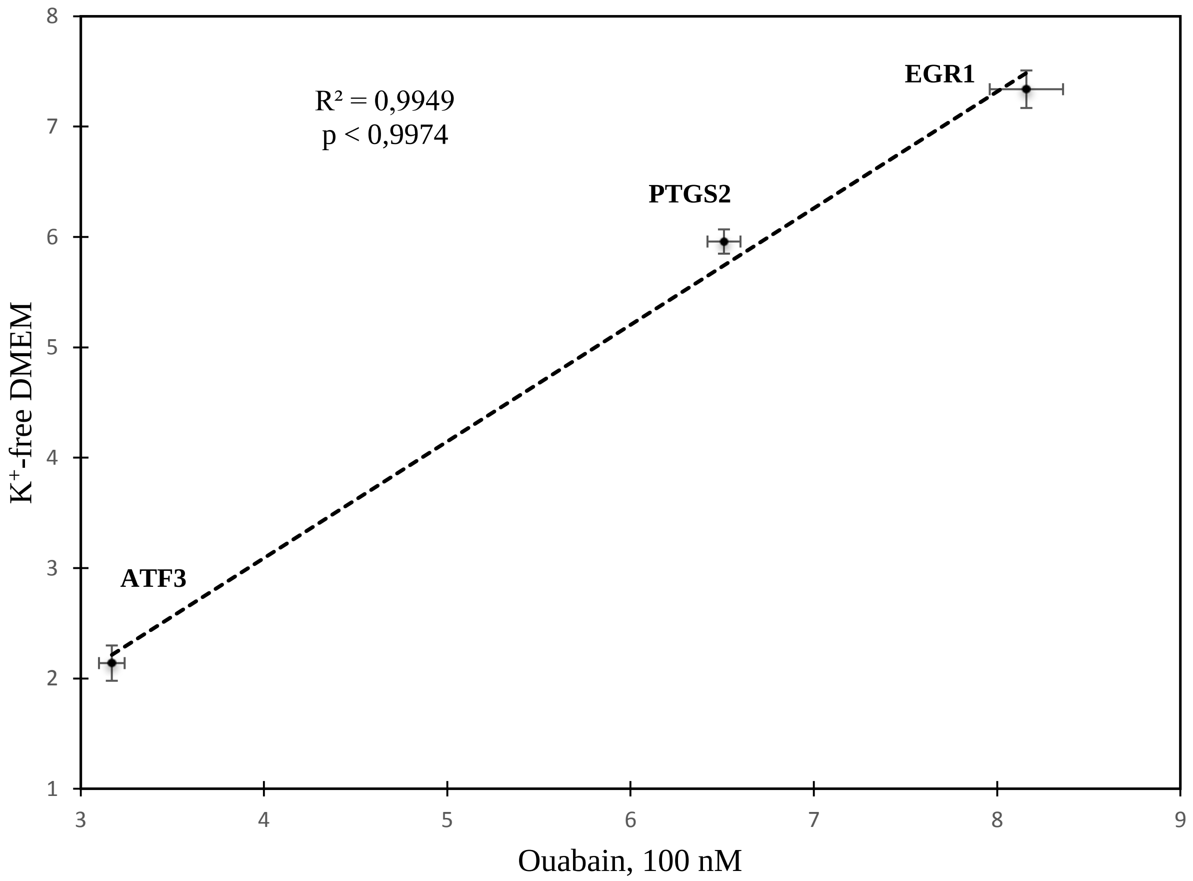

Figure S3. The positive correlation between gene expression (EGR1, PTGS2, and ATF3) in the presence of ouabain and in K+-free medium, $r_{xy} = 0,9974$ (r_{xy} is the Pearson correlation coefficient).

Ouabain, nM	Chromatin fragments, %			
	6 h	***24 h***	***48 h***	***72 h***
0	2.9±1.1	2.0±0.8	3.3±1.2	3.9±2.0
1	ND	ND	ND	3.0±1.6
3	ND	ND	ND	4.6±2.4
10	ND	ND	4.0±1.6	7.1±2.1
30	ND	3.1±0.9	19.5±4.4**	25.6±6.7**
100	4.5±1.8	15.0±4.7*	41.5±8.8$^{\#}$	36.7±7.0***
3,000	6.2.±2.1	43.4±7.3$^{\#}$	38.8±4.9$^{\#}$	35.4±5.5***

Table S1. Chromatin cleavage in HUVEC exposed to ouabain for 6, 24, 48, and 72 h. ND – not determined. The total content of ^{3}H-labeled DNA was taken as 100%. Means ± S.E. from experiments performed in quadruplicates are shown. *, **, ***, $^{\#}$ p<0.05, 0.02, 0.01 and 0.001 compared to ouabain-untreated cells.

Ouabain, nM	Caspase-3 activity *(nmol per mg of protein per h)*			
	6 h	***24 h***	***48 h***	***72 h***
0	0.25±0.04	0.23±0.02	0.29±0.06	0.37±0.05
1	ND	ND	ND	0.30±0.08
3	ND	ND	ND	0.36±0.04
10	ND	ND	0.40±0.03	0.65±0.18
30	ND	0.33±0.05	1.50±0.14*	2.06±0.18**
100	0.29±0.03	1.09±0.07*	3.15±0.28**	3.22±0.20**
3,000	0.44.±0.07	3.04±0.12**	2.88±0.29**	2.98±0.22**

Table S2. Caspase-3 activity in HUVEC exposed to ouabain for 6, 24, 48, and 72 h. ND – not determined. Means ± S.E. from experiments performed in triplicates are shown. *, ** p<0.01 and 0.001 compared to ouabain-untreated cells.

Additions, nM	Chromatin cleavage, %	Caspase-3 activity, nmol/mg prot/hr	Detached cells, %
None (control)	3.7±1.2	0.40±0.04	3.02±0.17
Ouabain, 1	3.4±0.8	0.32±0.06	2.82±0.20
Ouabain, 3	4.1±1.0	0.43±0.07	3.17±0.11
Ouabain, 10	13.4±3.2*	1.94±0.34*	34.62±3.37*
MBG, 10	3.4±1.4	0.29±0.05	3.78±0.22
MBG, 30	3.1±0.9	0.36±0.04	3.46±0.30

Table S3. Chromatin cleavage, caspase-3 activity and attachment of HUVEC exposed to ouabain and marinobufagenin (MBG) for 96 hr. Means ± S.E. from experiments performed in triplicates are shown. * p<0.01 compared to CTS-untreated cells.

Gene symbol	Fold of modulation	CYT	ENZ	GDD	IIR	TTR	UNK
EIF5	-2,84					■	
MIR216A	-2,08					■	
MIR27B	-2,02					■	
RPL27A	2,03					■	
C7orf53	2,05						■
KITLG	2,19	■		■			
C2orf61	2,2						■
GDF15	2,22			■			
MIR31	2,22					■	
JUNB	2,49					■	
ATF3	2,6					■	
ZFP36	3,77					■	
ADAMTS1	4,16		■				
PTGS2	5,86		■		■		
EGR1	5,87					■	
FOS	6,07					■	

Table S4. Functional classification of genes whose expression is modulated by 100 nM ouabain after 6-h incubation by more than 2-fold. **FC** – functional categories: **CYT** – cytokines; **ENZ** – unclassified enzymes; **GDD** – cell growth, differentiation, and death; **IIR** – immune and inflammatory responses; **TTR** – transcription/translation regulators; **UNK** – function remains unknown.

Gene symbol	Fold of activation	CYT	ENZ	GDD	IIR	TTR	ISG	OTH	UNK
IDI2	154,43		■						
KLF4	75,47					■			
FOSB	49,97					■			
OR2B6	42,6						■		
DSTNP2	41,24								■
BAMBI	29,56			■			■		
HIST1H2AH	27,72					■			
RPL13P5	24,64								■
HIST1H1T	22,87					■			
HIST1H4I	22,67					■			
RAB44	22,22								■
HIST1H2BJ	21,61					■			
SNORD77	19,45					■			
NR4A1	19,29			■	■	■			
RNASE10	18,79								■

**SNORD75**	18,76								
**LAG3**	18,75								
**NEFM**	18,24								
**ADAMTSL4**	17,98								
**C10orf110**	17								
**KRTAP5-2**	15,74								
**CENPL**	15,18								
**HIST1H2BG**	14,75								
**SNORA67**	14,42								
**LRRC23**	14,41								
**MAFB**	14,32								
**ARC**	13,69								
**SNORD50B**	13,57								
**NT5DC4**	13,35								
**HIST1H2AG**	12,9								
**GEM**	12,69								
**SNORD78**	12,65								
**HIST2H2BE**	12,48								
**EGR2**	12,47								
**HIST2H2BF**	12,39								
**NANOS1**	12,29								
**ID2**	12,01								
**EGR3**	11,87								
**IER2**	11,71								
**TLR7**	11,45								
**MIR503**	11,44								
**SNORA31**	11,11								
**SNORD44**	10,91								
**PEG10**	10,87								
**CDH16**	10,84								
**GFPT2**	10,61								
**RASL11A**	10,55								
**ACTN2**	10,18								
**RDH10**	10,14								
**ADM**	10,08								
**DNAJB1**	10,05								

Table S5. Functional classification of genes whose expression is activated by 3,000 nM ouabain after 6-h incubation by more than 10-fold. **FC** – functional categories: **CYT** – cytokines; **ENZ** – unclassified enzymes; **GDD** – cell growth, differentiation, and death; **IIR** – immune and inflammatory responses; **ISG** – intracellular signaling; **OTH** – others; **TRP** – transporters; **TTR** – transcription/translation regulators; **UNK** – function remains unknown.

Gene symbol	Fold of modulation	GDD	TTR	ISG	OTH	UNK
MIR181B1	-15,75					
MIR217	-13,45					
MIR221	-13,35					
LOC100130428	-11,24					
FLJ36840	-9,47					
SAMD9L	-9,44					
WEE1	-8,2					
PRO2012	-8,08					
DICER1	-7,4					
TRIM38	-7,38					
ZNF552	-6,97					
EDN1	-6,85					
MIRLET7A2	-6,23					
VCP	-6,14					
KIAA0430	-5,96					
ZNF192	-5,93					
ZFC3H1	-5,79					
MIR186	-5,79					
MIR222	-5,71					
AGGF1	-5,57					
PDGFB	-5,47					
KIAA0317	-5,46					
ZNF780B	-5,44					
LOC100287934	-5,42					
IREB2	-5,28					
RASSF2	-5,19					
ZNF330	-5,17					

Table S6. Functional classification of genes whose expression is inhibited after 6 h by 3,000 nM ouabain by more than 5-fold. **FC** – functional categories: **CYT** – cytokines; **ENZ** – unclassified enzymes; **GDD** – cell growth, differentiation and death; **IIR** – immune and inflammatory responses; **ISG** – intracellular signaling; **OTH** –others; **TRP** – transporters; **TTR** – transcription/translation regulators; **UNK** – function remains unknown. The table lists transcripts whose expression was decreased by more than 5-fold.

Gene symbol	3 nM ouabain, fold of modulation	30 nM MBG, fold of modulation	CYT	ENZ	GDD	IIR	TTR	ISG	TRP	OTH	UNK
FOS	33,28	13,29									
ZFP36	20,36	6,09									
EGR1	15,33	21,43									

ADAMTS1	13,04	5,01
PTGS2	11,36	9,02
EGR2	8,09	10,19
ATF3	7,39	8,13
ARRDC4	5,62	NC
NR4A1	5,39	7,29
NR4A3	5,38	6
STC1	5,35	NC
JUNB	5,06	1,53
KLF4	4,7	3,03
DUSP1	4,52	6,09
ADAMTS5	4,43	2,16
NR4A2	4,15	2,99
SNAI1	3,87	NC
HEY1	3,75	1,84
SOCS3	3,75	NC
VTRNA1-3	3,64	2,76
JUN	3,17	4,72
KITLG	3,05	1,44
C7orf53	3	1,33
EGR3	2,98	3,07
GDF15	2,98	NC
IDI2	2,87	NC
BAMBI	2,8	1,29
EFNB2	2,79	1,52
GEM	2,77	2,71
ABCG2	2,76	NC
SNORD3A	2,72	NC
FAM71A	2,66	1,7
IER2	2,65	1,54
HIST2H2BE	2,6	1,67
INSIG1	2,58	1,25
PROX1	2,52	2,7
HEY2	2,52	2,07
CA8	2,5	NC
HIST1H1T	2,48	1,32
TNFSF9	2,47	3,71
DLL4	2,46	NC
PTHLH	2,39	NC
APOLD1	2,36	NC
GUCY1B3	2,34	NC
IGFBP5	2,28	1,6
SPRY4	2,28	1,79
BCL6	2,27	NC

**HIST2H4A**	2,24	1,88
**KCNJ2**	2,24	2
**TSC22D2**	2,24	NC
**DDIT3**	2,23	2,78
**C2orf61**	2,23	NC
**HIST1H2BG**	2,19	NC
**HES1**	2,19	2,21
**ELMOD1**	2,18	NC
**MAFB**	2,16	1,89
**HIST1H2BD**	2,13	NC
**SNORD75**	2,11	-1,39
**ADAMTS9**	2,1	2,09
**ADAMTS4**	2,09	1,97
**KLF5**	2,07	1,62
**HIST2H2AA3**	2,05	NC
**NEFM**	2,04	-1,25
**PCDH17**	2,04	NC
**TXNIP**	2,03	2,22
**FJX1**	2,03	-1,33
**BHLHE40**	2,03	NC
**ID2**	2,02	1,73
**G0S2**	2,02	2,06
**IDI1**	2,01	1,21
**MIRLET7C**	-2,03	NC
**DTL**	-2,05	NC
**KIAA1370**	-2,07	1,22
**FAM111B**	-2,18	-1,59
**PDK4**	-2,32	-1,48
**TRA2A**	-2,4	NC
**EIF5**	-2,52	-2,59
**FBXO32**	-2,71	-1,73

Table S7. Functional classification of genes whose expression is modulated by 3 nM ouabain and 30 nM MBG after 96-h incubation. The table lists transcripts whose expression was changed in ouabain-treated cells by more than 2.0-fold. **FC** – functional categories: **CYT** – cytokines; **ENZ** – unclassified enzymes; **GDD –** cell growth, differentiation, and death; **IIR** – immune and inflammatory responses; **ISG** – intracellular signaling; **OTH** –others; **TRP** – transporters; **TTR** – transcription/translation regulators; **UNK** – function remains unknown. **NC** –expression of these genes in the presence of MBG was not changed.

Name	ID	C	O	E	R	rawP	adjP	Genes Symbol
biological process								
transmembrane receptor protein serine/threonine kinase signaling pathway	GO:0007178	255	4	0.19	21.15	2.40e-05	0.0026	EGR1; FOS; JUNB
decidualization	GO:0046697	17	2	0.01	158.60	6.70e-05	0.0026	PTGS2; JUNB
positive regulation of nitrogen compound metabolic process	GO:0051173	1151	6	0.85	7.03	5.94e-05	0.0026	KITLG; PTGS2; EGR1; FOS; ZFP36; JUNB
positive regulation of cellular metabolic process	GO:0031325	1801	7	1.34	5.24	6.23e-05	0.0026	KITLG; ATF3; PTGS2; EGR1; FOS; ZFP36; JUNB
developmental process involved in reproduction	GO:0003006	407	4	0.30	13.25	0.0001	0.0026	KITLG; PTGS2; ADAMTS1; JUNB
response to glucocorticoid stimulus	GO:0051384	136	3	0.10	29.74	0.0001	0.0026	PTGS2; FOS; JUNB
positive regulation of metabolic process	GO:0009893	1898	7	1.41	4.97	8.81e-05	0.0026	KITLG; ATF3; PTGS2; EGR1; FOS; ZFP36; JUNB
ovulation	GO:0030728	25	2	0.02	107.85	0.0001	0.0026	PTGS2; ADAMTS1
response to corticosterone stimulus	GO:0051412	24	2	0.02	112.34	0.0001	0.0026	FOS; JUNB
response to corticosteroid stimulus	GO:0031960	145	3	0.11	27.89	0.0001	0.0026	PTGS2; FOS; JUNB
molecular function								
structure-specific DNA binding	GO:0043566	179	3	0.13	23.46	0.0002	0.0034	EGR1; FOS; JUNB
double-stranded DNA binding	GO:0003690	139	3	0.10	30.21	0.0001	0.0034	EGR1; FOS; JUNB
sequence-specific DNA binding	GO:0043565	625	4	0.45	8.96	0.0007	0.0079	ATF3; EGR1; FOS; JUNB

sequence-specific DNA binding transcription factor activity	GO:0003700	938	4	0.67	5.97	0.0030	0.0176	ATF3; EGR1; FOS; JUNB
nucleic acid binding transcription factor activity	GO:0001071	940	4	0.67	5.96	0.0031	0.0176	ATF3; EGR1; FOS; JUNB
protein dimerization activity	GO:0046983	915	4	0.65	6.12	0.0028	0.0176	ATF3; PTGS2; FOS; JUNB
growth factor activity	GO:0008083	150	2	0.11	18.66	0.0049	0.0238	KITLG; GDF15
transcription corepressor activity	GO:0003714	180	2	0.13	15.55	0.0069	0.0291	ATF3; JUNB
cytokine activity	GO:0005125	190	2	0.14	14.73	0.0077	0.0291	KITLG; GDF15
nucleic acid binding	GO:0003676	2978	6	2.13	2.82	0.0087	0.0296	ATF3; EIF5; EGR1; FOS; ZFP36; JUNB
cellular component								
intracellular non-membrane-bounded organelle	GO:0043232	3551	5	2.53	1.98	0.0844	0.3693	KITLG; ATF3; FOS; ZFP36; JUNB
extracellular region part	GO:0044421	1021	3	0.73	4.13	0.0317	0.3693	KITLG; GDF15; ADAMTS1
membrane-enclosed lumen	GO:0031974	3193	5	2.27	2.20	0.0568	0.3693	ATF3; PTGS2; EGR1; FOS; JUNB
organelle lumen	GO:0043233	3151	5	2.24	2.23	0.0540	0.3693	ATF3; PTGS2; EGR1; FOS; JUNB
intracellular organelle lumen	GO:0070013	3106	5	2.21	2.26	0.0511	0.3693	ATF3; PTGS2; EGR1; FOS; JUNB
non-membrane-bounded organelle	GO:0043228	3551	5	2.53	1.98	0.0844	0.3693	KITLG; ATF3; FOS; ZFP36; JUNB
nucleolus	GO:0005730	1448	3	1.03	2.91	0.0764	0.3693	ATF3; FOS; JUNB
nucleoplasm	GO:0005654	1400	4	1.00	4.02	0.0131	0.3693	ATF3; EGR1; FOS; JUNB
nuclear lumen	GO:0031981	2583	4	1.84	2.18	0.0960	0.3733	ATF3; EGR1; FOS; JUNB
extracellular space	GO:0005615	795	2	0.57	3.54	0.1067	0.3735	KITLG; GDF15

Table S8. Gene ontology analysis of genes whose expression is modulated by 100 nM ouabain after 6-h incubation by more than 2-fold.

Name	ID	C	O	E	R	rawP	adjP	Genes Symbol
biological process								
regulation of cell differentiation	GO:0045595	957	24	4.40	5.45	1.45e-12	1.58e-09	EFNB2; HES1; DLL4; PTHLH; SNAI1; PROX1; MAFB; ID2; SOCS3; HEY2; JUN; NEFM; PTGS2; APOLD1; INSIG1; BAMBI; KLF4; ZFP36; IGFBP5; JUNB; BCL6; KITLG; ADAMTS9; HEY1;
regulation of developmental process	GO:0050793	1365	26	6.28	4.14	6.26e-11	3.41e-08	EFNB2; HES1; DLL4; EGR1; PTHLH; SNAI1; PROX1; MAFB; ID2; SOCS3; HEY2; JUN; PTGS2; NEFM; APOLD1; INSIG1; BAMBI; KLF4; ZFP36; IGFBP5; JUNB; BCL6; KITLG; ADAMTS9; DDIT3; HEY1
circulatory system development	GO:0072359	728	19	3.35	5.67	3.11e-10	5.64e-08	EFNB2; HES1; DLL4; EGR1; SNAI1; NR4A1; PROX1; ID2; EGR3; ADAMTS1; SOCS3; HEY2; JUN; PTGS2; APOLD1; KLF4; JUNB; KLF5; HEY1
anatomical structure formation involved in morphogenesis	GO:0048646	1546	27	7.11	3.80	1.64e-10	5.64e-08	EFNB2; HES1; DLL4; PTHLH; SNAI1; NR4A1; PROX1; MAFB; ID2; EGR3; ADAMTS1; EGR2; SOCS3; HEY2; JUN; PTGS2; NEFM; APOLD1; INSIG1; KLF4; IGFBP5; JUNB; NR4A3; NR4A2; KLF5; HEY1; DUSP1
cardiovascular system development	GO:0072358	728	19	3.35	5.67	3.11e-10	5.64e-08	EFNB2; HES1; DLL4; EGR1; SNAI1; NR4A1; PROX1; ID2; EGR3; ADAMTS1; SOCS3; HEY2; JUN; PTGS2; APOLD1; KLF4; JUNB; KLF5;

								HEY1
blood vessel development	GO:0001568	478	16	2.20	7.28	2.96e-10	5.64e-08	EFNB2; HES1; DLL4; EGR1; NR4A1; PROX1; EGR3; SOCS3; HEY2; JUN; PTGS2; APOLD1; KLF4; JUNB; KLF5; HEY1
vasculature development	GO:0001944	502	16	2.31	6.93	6.05e-10	9.41e-08	EFNB2; HES1; DLL4; EGR1; NR4A1; PROX1; EGR3; SOCS3; HEY2; JUN; PTGS2; APOLD1; KLF4; JUNB; KLF5; HEY1
anatomical structure morphogenesis	GO:0009653	1982	29	9.12	3.18	1.69e-09	2.30e-07	EFNB2; HES1; DLL4; PTHLH; SNAI1; NR4A1; PROX1; MAFB; ID2; EGR3; ADAMTS1; EGR2; SOCS3; HEY2; JUN; PTGS2; NEFM; APOLD1; INSIG1; BAMBI; KLF4; IGFBP5; JUNB; NR4A3; BCL6; NR4A2; KLF5; HEY1; DUSP1
transcription from RNA polymerase II promoter	GO:0006366	1356	24	6.24	3.85	2.11e-09	2.55e-07	HES1; DLL4; EGR1; SNAI1; NR4A1; PROX1; MAFB; ID2; BHLHE40; FOS; EGR2; HEY2; JUN; INSIG1; KLF4; ZFP36; TXNIP; JUNB; NR4A3; BCL6; NR4A2; KLF5; DDIT3; HEY1
positive regulation of nitrogen compound metabolic process	GO:0051173	1151	22	5.29	4.16	3.13e-09	3.41e-07	HES1; EGR1; PTHLH; SNAI1; PROX1; MAFB; ID2; FOS; EGR2; HEY2; JUN; PTGS2; INSIG1; BAMBI; KLF4; ZFP36; JUNB; KITLG; NR4A2; KLF5; DDIT3; HEY1
molecular function								
sequence-specific DNA binding transcription factor activity	GO:0003700	938	22	4.09	5.38	2.12e-11	1.25e-09	HES1; EGR1; PTHLH; SNAI1; PROX1; MAFB; ID2; FOS; EGR2; HEY2; JUN; PTGS2; INSIG1; BAMBI; KLF4;

								ZFP36; JUNB; KITLG; NR4A2; KLF5; DDIT3; HEY1
nucleic acid binding transcription factor activity	GO:0001071	940	22	4.10	5.37	2.21e-11	1.25e-09	HES1; EGR1; PTHLH; SNAI1; PROX1; MAFB; ID2; FOS; EGR2; HEY2; JUN; PTGS2; INSIG1; BAMBI; KLF4; ZFP36; JUNB; KITLG; NR4A2; KLF5; DDIT3; HEY1
sequence-specific DNA binding RNA polymerase II transcription factor activity	GO:0000981	227	12	0.99	12.13	2.14e-10	8.06e-09	JUN; HEY2; HES1; EGR1; KLF4; SNAI1; NR4A1; NR4A3; PROX1; NR4A2; BHLHE40; HEY1
sequence-specific DNA binding	GO:0043565	625	17	2.72	6.24	7.70e-10	2.18e-08	HES1; EGR1; SNAI1; NR4A1; PROX1; MAFB; ATF3; FOS; HEY2; JUN; KLF4; JUNB; BCL6; NR4A3; NR4A2; DDIT3; HEY1
RNA polymerase II core promoter proximal region sequence-specific DNA binding transcription factor activity	GO:0000982	80	7	0.35	20.08	5.19e-08	1.17e-06	HES1; BHLHE40; EGR1; KLF4; SNAI1; NR4A1; NR4A3;
protein dimerization activity	GO:0046983	915	17	3.99	4.26	2.18e-07	4.11e-06	HES1; NR4A1; ABCG2; ID2; HIST2H2BE; ATF3; BHLHE40; FOS; HEY2; JUN; PTGS2; GUCY1B3; JUNB; NR4A2; HIST1H2BD; DDIT3; HEY1
DNA binding	GO:0003677	2123	25	9.25	2.70	9.72e-07	1.57e-05	HES1; EGR1; SNAI1; NR4A1; PROX1; MAFB; HIST2H2BE; ATF3; EGR3; BHLHE40; FOS; EGR2; HEY2; JUN; HIST1H1T; KLF4; ZFP36; JUNB; NR4A3; BCL6; KLF5; NR4A2; HIST1H2BD; DDIT3; HEY1
RNA polymerase II activating transcription	GO:0001102	25	4	0.11	36.71	3.86e-06	5.39e-05	JUN; HEY2; BHLHE40; EGR2

factor binding								
RNA polymerase II transcription factor binding	GO:0001085	56	5	0.24	20.49	4.29e-06	5.39e-05	JUN; HEY2; BHLHE40; KLF4; EGR2
isopentenyl-diphosphate delta-isomerase activity	GO:0004452	2	2	0.01	229.44	1.87e-05	0.0002	IDI2; IDI1
cellular component								
chromatin	GO:0000785	272	8	1.09	7.34	1.23e-05	0.0009	JUN; HEY2; HIST1H1T; KLF4; JUNB; ID2; HIST2H2BE; HIST1H2BD
chromosomal part	GO:0044427	497	9	1.99	4.52	0.0002	0.0070	JUN; HEY2; HIST1H1T; KLF4; JUNB; BCL6; ID2; HIST2H2BE; HIST1H2BD
chromosome	GO:0005694	590	9	2.36	3.81	0.0005	0.0117	JUN;HEY2;HIST1H1T;KLF4;JUNB;BCL6;ID2;HIST2H2BE;HIST1H2BD
transcription factor complex	GO:0005667	269	6	1.08	5.57	0.0007	0.0123	MAFB; JUN; FOS; KLF4; NR4A1; NR4A3
nucleus	GO:0005634	5499	34	22.04	1.54	0.0014	0.0163	EGR1; PTHLH; ID2; HIST2H2BE; EGR3; BHLHE40; DTL; HEY2; JUN; FBXO32; HIST1H1T; ZFP36; TXNIP; JUNB; BCL6; HIST1H2BD; DUSP1; HES1; NR4A1; SNAI1; MAFB; PROX1; ATF3; FOS; EGR2; PTGS2; ADAMTS5; KLF4; NR4A3; KLF5; NR4A2; GEM; DDIT3; HEY1
nucleosome	GO:0000786	52	3	0.21	14.39	0.0012	0.0163	HIST2H2BE; HIST1H1T; HIST1H2BD
nuclear lumen	GO:0031981	2583	20	10.35	1.93	0.0019	0.0171	EGR1; PTHLH; NR4A1; MAFB; ID2; ATF3; BHLHE40; FOS; HEY2; JUN; ADAMTS5; HIST1H1T; KLF4; JUNB; NR4A3; BCL6; NR4A2; KLF5; DDIT3; DUSP1;
extracellular region part	GO:0044421	1021	11	4.09	2.69	0.0022	0.0171	ADAMTS5; PTHLH; ADAMTS4; IGFBP5; FJX1; KITLG; GDF15;

								ADAMTS1; ADAMTS9; TNFSF9; STC1
membrane-enclosed lumen	GO:0031974	3193	23	12.80	1.80	0.0021	0.0171	EGR1; PTHLH; NR4A1; PDK4; MAFB; ID2; ATF3; BHLHE40; FOS; HEY2; JUN; PTGS2; ADAMTS5; HIST1H1T; KLF4; TXNIP; JUNB; NR4A3; BCL6; NR4A2; KLF5; DDIT3; DUSP1
nuclear part	GO:0044428	2892	21	11.59	1.81	0.0032	0.0198	EGR1; PTHLH; NR4A1; MAFB; ID2; ATF3; BHLHE40; FOS; DTL; HEY2; JUN; ADAMTS5; HIST1H1T; KLF4; JUNB; NR4A3; BCL6; NR4A2; KLF5; DDIT3; DUSP1;

Table S9. Gene ontology analysis of genes whose expression is modulated by 3 nM ouabain and 30 nM MBG after 96-h incubation. The table lists transcripts whose expression was changed in ouabain-treated cells by more than 2.0-fold.

**Name**	**ID**	**C**	**O**	**E**	**R**	**rawP**	**adjP**	**Genes Symbol**
biological process								
chromatin assembly	GO:0031497	93	4	0.21	19.33	5.07e-05	0.0042	HIST2H2BE; HIST1H2AH; HIST1H2BJ;
cell migration involved in sprouting angiogenesis	GO:0002042	24	3	0.05	56.17	1.95e-05	0.0042	EGR3; KLF4; NR4A1
nucleosome assembly	GO:0006334	84	4	0.19	21.40	3.40e-05	0.0042	HIST2H2BE; HIST1H2AH; HIST1H2BJ; HIST1H1T
brain segmentation	GO:0035284	5	2	0.01	179.75	4.77e-05	0.0042	MAFB; EGR2
central nervous system segmentation	GO:0035283	5	2	0.01	179.75	4.77e-05	0.0042	MAFB; EGR2
rhombomere 5 development	GO:0021571	4	2	0.01	224.68	2.86e-05	0.0042	MAFB; EGR2

sprouting angiogenesis	GO:0002040	43	3	0.10	31.35	0.0001	0.0045	EGR3; KLF4; NR4A1
chromatin assembly or disassembly	GO:0006333	114	4	0.25	15.77	0.0001	0.0045	HIST2H2BE; HIST1H2AH; HIST1H2BJ; HIST1H1T
nucleosome organization	GO:0034728	104	4	0.23	17.28	7.85e-05	0.0045	HIST2H2BE; HIST1H2AH; HIST1H2BJ; HIST1H1T
rhombomere development	GO:0021546	8	2	0.02	112.34	0.0001	0.0045	MAFB; EGR2
molecular function								
protein dimerization activity	GO:0046983	915	7	2.03	3.45	0.0033	0.0544	HIST2H2BE; ID2; HIST1H2AH; HIST1H2BJ; ACTN2; NR4A1; FOSB
nucleic acid binding	GO:0003676	2978	14	6.60	2.12	0.0025	0.0544	NANOS1; TLR7; HIST1H1T; KLF4; NR4A1; FOSB; HIST2H2BE; MAFB; HIST1H2AH; EGR3; HIST1H2BJ; PEG10; RNASE10; EGR2
heterocyclic compound binding	GO:1901363	4938	18	10.94	1.65	0.0079	0.0544	RAB44; NANOS1; RASL11A; NR4A1; RDH10; HIST2H2BE; MAFB; HIST1H2AH; HIST1H2BJ; EGR3; RNASE10; EGR2; TLR7; HIST1H1T; KLF4; FOSB; GEM; PEG10
RNA polymerase II transcription regulatory region sequence-specific DNA binding transcription factor activity involved in positive regulation of	GO:0001228	58	2	0.13	15.57	0.0073	0.0544	KLF4; NR4A1

transcription								
protein heterodimerization activity	GO:0046982	344	4	0.76	5.25	0.0067	0.0544	HIST2H2BE; HIST1H2AH; HIST1H2BJ; NR4A1
RNA polymerase II core promoter proximal region sequence-specific DNA binding transcription factor activity involved in positive regulation of transcription	GO:0001077	54	2	0.12	16.72	0.0063	0.0544	KLF4; NR4A1
ion channel binding	GO:0044325	38	2	0.08	23.76	0.0032	0.0544	ID2; ACTN2
RNA polymerase II transcription factor binding	GO:0001085	56	2	0.12	16.12	0.0068	0.0544	KLF4; EGR2
DNA binding	GO:0003677	2123	11	4.70	2.34	0.0043	0.0544	HIST1H1T; KLF4; NR4A1; FOSB; HIST2H2BE; MAFB; HIST1H2AH; EGR3; HIST1H2BJ; PEG10; EGR2
organic cyclic compound binding	GO:0097159	4988	18	11.05	1.63	0.0089	0.0552	RAB44; NANOS1; RASL11A; NR4A1; RDH10; HIST2H2BE; MAFB; HIST1H2AH; HIST1H2BJ; EGR3; RNASE10; EGR2; TLR7; HIST1H1T; KLF4; FOSB; GEM; PEG10
cellular component								
nucleosome	GO:0000786	52	4	0.11	37.19	3.81e-06	0.0002	HIST2H2BE; HIST1H2AH; HIST1H2BJ; HIST1H1T
chromatin	GO:0000785	272	6	0.56	10.66	1.73e-05	0.0006	HIST2H2BE; ID2; HIST1H2AH; HIST1H2BJ; HIST1H1T; KLF4

chromosomal part	GO:0044427	497	7	1.03	6.81	5.67e-05	0.0009	HIST2H2BE; ID2; HIST1H2AH; HIST1H2BJ; HIST1H1T; KLF4; CENPL
protein-DNA complex	GO:0032993	94	4	0.19	20.57	4.04e-05	0.0009	HIST2H2BE; HIST1H2AH; HIST1H2BJ; HIST1H1T
chromosome	GO:0005694	590	7	1.22	5.74	0.0002	0.0026	HIST2H2BE; ID2; HIST1H2AH; HIST1H2BJ; HIST1H1T; KLF4; CENPL
transcription factor complex	GO:0005667	269	3	0.56	5.39	0.0178	0.1928	MAFB; KLF4; NR4A1
nucleus	GO:0005634	5499	17	11.38	1.49	0.0312	0.2689	RASL11A; NR4A1; RDH10; MAFB; ID2; HIST2H2BE; HIST1H2AH; DNAJB1; EGR3; HIST1H2BJ; EGR2; CENPL; HIST1H1T; KLF4; FOSB; GEM; PEG10
intracellular non-membrane-bounded organelle	GO:0043232	3551	12	7.35	1.63	0.0455	0.2689	NEFM; HIST1H1T; RASL11A; KLF4; ID2; HIST2H2BE; HIST1H2AH; ARC; DNAJB1; ACTN2; HIST1H2BJ; CENPL
dendritic spine	GO:0043197	156	2	0.32	6.20	0.0411	0.2689	ARC; ACTN2
neuron spine	GO:0044309	156	2	0.32	6.20	0.0411	0.2689	ARC; ACTN2

Table S10. Gene ontology analysis of genes whose expression is activated by 3,000 nM ouabain after 6-h incubation by more than 10-fold.

Name	ID	C	O	E	R	rawP	adjP	Genes Symbol
biological process								
positive regulation of mitosis	GO:0045840	34	2	0.04	52.87	0.0006	0.0421	PDGFB; EDN1
positive regulation of nuclear division	GO:0051785	34	2	0.04	52.87	0.0006	0.0421	PDGFB; EDN1
negative regulation of blood coagulation	GO:0030195	37	2	0.04	48.58	0.0008	0.0421	PDGFB; EDN1
regulation of odontogenesis	GO:0042481	24	2	0.03	74.89	0.0003	0.0421	DICER1; EDN1
M phase	GO:0000279	510	5	0.57	8.81	0.0002	0.0421	WEE1; KIAA0430; PDGFB; DICER1; EDN1
positive regulation of smooth muscle cell proliferation	GO:0048661	40	2	0.04	44.94	0.0009	0.0421	PDGFB; EDN1
positive regulation of endothelial cell migration	GO:0010595	40	2	0.04	44.94	0.0009	0.0421	PDGFB; EDN1
negative regulation of coagulation	GO:0050819	40	2	0.04	44.94	0.0009	0.0421	PDGFB; EDN1
cell cycle	GO:0007049	1334	6	1.48	4.04	0.0021	0.0453	RASSF2; WEE1; KIAA0430; PDGFB; DICER1; EDN1
molecular function								
eukaryotic cell surface binding	GO:0043499	23	2	0.02	81.14	0.0003	0.0117	AGGF1; PDGFB
cell surface binding	GO:0043498	53	2	0.06	35.21	0.0014	0.0273	AGGF1; PDGFB
cation binding	GO:0043169	3698	8	3.96	2.02	0.0240	0.2340	WEE1; IREB2; TRIM38; ZNF192; ZNF780B; ZNF330; DICER1; ZFC3H1
metal ion binding	GO:0046872	3599	8	3.86	2.07	0.0204	0.2340	WEE1; IREB2; TRIM38; ZNF192; ZNF780B; ZNF330; DICER1; ZFC3H1
ATPase activity	GO:0016887	334	2	0.36	5.59	0.0486	0.3159	VCP; DICER1

RNA binding	GO:0003723	760	3	0.81	3.68	0.0445	0.3159	KIAA0430; IREB2; DICER1
ATP binding	GO:0005524	1395	3	1.50	2.01	0.1827	0.3312	WEE1; VCP; DICER1
nucleic acid binding	GO:0003676	2978	6	3.19	1.88	0.0789	0.3312	KIAA0430; AGGF1; IREB2; ZNF192; ZNF780B; DICER1
purine ribonucleoside binding	GO:0032550	1719	3	1.84	1.63	0.2774	0.3312	WEE1; VCP; DICER1
purine nucleoside binding	GO:0001883	1722	3	1.85	1.63	0.2783	0.3312	WEE1; VCP; DICER1
cellular component								
intracellular part	GO:0044424	####	13	11.71	1.11	0.3418	0.7200	KIAA0430; WEE1; IREB2; AGGF1; ZNF192; ZNF780B; ZNF330; DICER1; EDN1; RASSF2; KIAA0317; PDGFB; VCP
cytoplasm	GO:0005737	8531	11	8.82	1.25	0.2008	0.7200	KIAA0430; WEE1; IREB2; AGGF1; ZNF192; DICER1; EDN1; RASSF2; KIAA0317; PDGFB; VCP
cell	GO:0005623	####	15	13.90	1.08	0.3600	0.7200	KIAA0430; WEE1; IREB2; AGGF1; TRIM38; ZNF192; ZNF780B; ZNF330; DICER1; ZFC3H1; EDN1; RASSF2; KIAA0317; PDGFB; VCP
intracellular	GO:0005622	####	15	12.00	1.25	0.0632	0.7200	KIAA0430; WEE1; IREB2; AGGF1; TRIM38; ZNF192; ZNF780B; ZNF330; DICER1; ZFC3H1; EDN1; RASSF2; KIAA0317; PDGFB; VCP
chromosomal part	GO:0044427	497	2	0.51	3.89	0.0919	0.7200	ZNF330; VCP
nucleolus	GO:0005730	1448	3	1.50	2.00	0.1839	0.7200	ZNF192; ZNF330; VCP
nuclear lumen	GO:0031981	2583	4	2.67	1.50	0.2720	0.7200	WEE1; ZNF192; ZNF330; VCP
chromosome	GO:0005694	590	2	0.61	3.28	0.1226	0.7200	ZNF330; VCP

membrane-enclosed lumen	GO:0031974	3193	5	3.30	1.51	0.2213	0.7200	WEE1; PDGFB; ZNF192; ZNF330; VCP
nuclear part	GO:0044428	2892	4	2.99	1.34	0.3498	0.7200	WEE1; ZNF192; ZNF330; VCP

Table S11. Gene ontology analysis of genes whose expression is inhibited after 6 h by 3,000 nM ouabain by more than 5- fold.
